# Resection of Gasserian Ganglion and Extirpation of Trigeminus*From original text in *Le Progre’s Medical*.

**Published:** 1896-10

**Authors:** Paul Poirier

**Affiliations:** Paris


### RESECTION OF GASSERIAN GANGLION AND EXTIRPATION OF TRIGEMINUS.*

BY DR. PAUL POIRIER, PARIS.

Translated from the French by G. M. Corput, Atlanta, Ga.

Having been called on to make a resection of the Gasserian ganglion on a patient in the service of Professor Raymond, I began by studying and repeating the different operations now in vogue on the cadaver.

After many experiments, I arrived at a fixed procedure in this operation, which many surgeons declare very difficult, if not impossible.

After having performed the operation twenty-five times on the cadaver in the amphitheatre of the School of Practice, and in Professor Berger's amphitheatre on the living subject, I do not hesitate to declare that to the surgeon with an ordinary knowledge of anatomy, the resection of the Gasserian ganglion and extirpation of the trigeminus present no real difficulties.

*From original text in *Le Progre's Medical.*

To understand and execute this operation with security and success, it is indispensable to have well in mind the principal points of the anatomy of the temporal region, the pterygo—maxillary fossa and middle fossa of the skull.

The method of procedure which I have adopted, after many trials, has really nothing original about it, unless it be the division and minute description of the various steps of the operation, which simplify and modify it somewhat, it having been borrowed from the operations of Rose, Horsley, Krause, Doyen and others.

However, I have been unable to find the last step of this operation described by any of the authors whose works I have read.

### FIRST STEP.

The cutaneous incision begins at the tuberosity of the malar bone and goes up in a vertical manner to the junction of the orbital process of the malar and frontal bones; here it turns to cross the temporal region in a horizontal manner, and again turns to descend vertically in the pre-auricular furrow to the tragus, forming an inverted U, with the anterior branch a little longer than the posterior one. This differs but little from the line of incision which Solzer advises for the resection of the inferior maxillary nerve in the foramen ovale, except that he, and those who imitated him, divided in the same line the skin, fascia and temporal muscle.

Incise freely down to the malar bone, more carefully in the temporal region, and especially so in descending in the pre-auricular furrow, in order to control, as long as possible, the superficial temporal vessels, which are to be ligated and divided at the auricular angle of the flap.

The dissection of the flap first divides the malar insertions of the zygomaticus major muscle, uncovers the temporal fascia, the zygomatic process, and one centimeter below this the superior lobes of the parotid gland, which it is important to spare.

Do not be disturbed by the slight hemorrhage, which occurs during the dissection of the flap, resulting from the division of the superficial temporal arteries and veins; this will be controlled when the vessel is ligated at the auricular angle of the flap.

### SECOND STEP.

The flap having been dissected and turned back towards the angle of the jaw, incise, as in the operations of Kronlein and Rose, the temporal fascia along the orbital process, and the zygoma, but two or three millimeters from the osseous border, in order to be able to take sutures after the operation.

Carry this incision well back. Here the middle temporal artery will have to be ligated and divided; after this the temporal muscle will bleed less; next pass a Nélaton grooved director under the external orbital process and work it under the malar bone, letting its point emerge at the level of the malar tubercle; a small incision in the tendinous insertion of the masseter muscle will facilitate the passage of the sound.

Leave the sound in place and divide the bone over it with a small metacarpal saw. This manœuver is very simple, as the sound protects the skin and adjacent tissues.

Next, with the bone-cutting forceps, divide the zygomatic process at the level of the point of junction of its two roots, cutting, in an oblique direction, from just behind the zygomatic tubercle, to a point just in front of the condyle of the inferior maxilla.

Do not lose sight of the fact that the foramen ovale, towards which you are cutting, is situated at the base of the transverse root of the zygomatic process, about thirty-five millimeters from the zygomatic tubercle.

By this method, besides having a larger opening, a surface of section is obtained double that of a transverse cut, an advantage not to be overlooked in the osseous union to come.

Be careful not to cut too deep with the point of the forceps, otherwise the upper synovial sac of the temporo-maxillary articulation may be opened, which, though not a grave accident, is better avoided.

The width of the osseous breech thus obtained is about four centimeters; in one of my subjects, however, it was only about thirty-five millimeters, in which case the resection of the ganglion was much more difficult.

The bone having been divided, the zygomatic arch must be turned back. To do this, turn out the bony arch, and with the grooved director carefully separate the temporal and masseter muscles from each other; during this separation an arteriole and some tolerably large veins will be encountered; this is not the masseteric artery, which passes lower down, in the sigmoid notch. Carry this separation far enough down to easily outline the coronoid process of the inferior maxilla, which is surrounded by the tendon of the temporal muscle. Pass the sound along the anterior and posterior borders of the temporal muscle. Along the posterior border the masseteric vessels and nerve are found. First, free the anterior border of the muscle from the soft fat which surrounds it, and push it down towards the jaw; it is useless to remove this fat, as it can be pushed well out of the way and later on it helps to fill the vast excavation which will have been made.

THIRD STEP.

The borders of the temporal muscle having been separated from adjacent tissues, and the coronoid process having been well outlined, divide the summit of this process with the bone-cutting forceps. As the temporal muscle descends quite low on the internal face of this process, the division of the tendon and the inferior fibres of the muscle will have to be completed with the knife. When the inferior end of the muscle has been entirely divided, carefully turn it back towards the temporal fossa. Sometimes considerable difficulty is experienced in separating this muscle from the external pterygoid muscle, as the interstice between these muscles is often traversed by an arteriole and several veins, quite often, also, by the internal maxillary artery. In either case, carefully ligate the artery and veins. It is of much advantage if it be the internal maxillary, as the remainder of the operation will be much easier and the wound almost bloodless. When this has been done, turn back the temporal muscle and denude the temporal fossa, with the handle of a scalpel, from the spinous process of the sphenoid upwards for about the width of two fingers.

FOURTH STEP.

Remember that the spheno-temporal region is almost horizontal, and that the foramen ovale is at a depth of about twenty to twenty-three millimeters, on the transverse root of the zygomatic process (temporal condyle). Denude the spheno-tempora region with the curved periosteotome from the spinous proces of the sphenoid. Work the instrument between the periosteum and the bones in a transverse direction to immediately in front of the temporal condyle. The back of the instrument pushes back and protects the external pterygoid and vessels.

After sponging the cavity out well, with a compress held in place for a few seconds, the posterior border of the pterygoid process of the sphenoid will be seen and recognized, and immediately behind it the foramen ovale, from which emerges a bundle of reddish tissue, the inferior maxillary nerve. With the blunt point of the grooved director isolate the nervo-vascular bundle from the adjacent tissues.

There are three points which are of much importance in this step, as I have said. You will see and recognize—for you must see and recognize—first, the posterior border of the external wing of the pterygoid process of the sphenoid; second, the inferior maxillary nerve emerging from the foramen ovale; third, do not trust to the sense of touch in these points, for here, as in everything else, the eye is the best guide.

### FIFTH STEP.

Most other authors recommend that, at this point of the operation, a trephine be used to make the opening in the bone, either in the temporal fossa, or in the spheno-temporal region. It is, however, much easier, and less dangerous, and requires less time to make the opening, with the bone-cutting chisel, in the temporal fossa, as the bone is of unequal thickness and is, just at this point, crossed by the middle meningeal artery, which is sometimes enclosed in a complete bony canal. Up to the present time, I have done sixty-four trepanations, and I have never used a trephine. I use a sharp chisel, with a good strong handle, which I have had made by M. Collin, instrument maker, Paris.

Some authors claim that there is great danger of contusion by this method of opening the cranium; this assertion is, however, not supported by proofs. Never yet have I seen any evidence of cerebral shock; in fact, in several cases of injuries, in which the patient was in a state of coma at the moment of intervention, he recovered consciousness immediately afterwards.

Therefore, with a sharp chisel, with a good handle so that it can be firmly held in the hand, applied almost parallel to the osseous surface, outline an opening circular in shape and about two centimeters in diameter. With light strokes of the mallet make the chisel penetrate the outer table of the skull, giving it a prizing motion to elevate the disc of bone; continue this maneuver until a fragment of the entire thickness of the osseous wall, very thin at this point, has been removed.

The dura mater being exposed, separate it from the osseous surface with a suitable instrument; then enlarge the opening in the temporal fossa with the gouge forceps; cut away the sub-temporal floor with the same instrument, until the foramen ovale is reached; this is also opened with the gouge forceps.

It goes without saying that the dura mater is to be first separated from the bone. Usually, an opening in the bone three centimeters in diameter is large enough; however, it can made larger, if necessary.

SIXTH STEP.

The inferior maxillary nerve having been recognized and the foramen ovale hollowed out, the large nerve is seen, the cellular envelope of which is apparently continuous with the dura mater, which covers the temporo-sphenoidal lobe. With a malleable retractor, to which the proper curve has been given, carefully raise the temporo-sphenoidal lobe; then, while the retractor is held by the left hand, carefully introduce a blunt-pointed grooved director into the groove formed by the junction of the inferior maxillary and the dura mater, and by careful manipulation the latter is easily detached; raise the dura mater with the retractor, as it is separated from the ganglion by the grooved director, and in a few seconds the cerebral surface of the ganglion will be exposed.

The superior maxillary nerve is then easily recognized, and probably the ophthalmic branch also, passing along the external wall of the cavernous sinus. The cranial face of the ganglion must be disengaged in the same manner. Then, with a blunt-pointed neurotome, divide the inferior maxillary nerve in the foramen ovale, which has now become a large groove. Be careful to cut from behind forward, introducing the neurotome between the middle meningeal artery and the nerve, in order to avoid wounding the artery.

Then divide in the same manner, but inside the cranial cavity, the superior maxillary nerve; seize the central end of the inferior maxillary nerve with a dissecting forceps, and raising the ganglion by this nerve, separate it from the petrous portion of the temporal bone, up to the depression for the ganglion of Meckel.

Here, as on the cerebral surface, be careful not to carry the separation too far in towards the cavernous sinus. Be very careful in this, as the ganglion rests on the internal carotid artery at this point, and is only separated from it by a thin fibrous layer.

The ganglion being thus detached, and visible from all its surfaces, seize the trifacial with a pair of ordinary artery forceps, at the level of the point where it enters the ganglion. Do not pull upon it at first, but twist it, in order to tear it out at its origin. When the trunk of the trifacial has been torn out, continue the torsion, to remove, by tearing, from behind forwards, the ophthalmic branch.

Do not forget that ordinarily the ophthalmic branch is not separable from the walls of the cavernous sinus. Should it be desirable to remove this branch, the sinus is invariably opened. Without attaching too much importance to this accident (for the cavernous sinus is not a large one, and will not bleed very abundantly), I advise that entire removal of the ophthalmic branch be not attempted. It is usually torn through where it emerges from the ganglion, and as that is all that is required, I do not see the necessity of attempting more.

The operation, as I describe it, requires about fifteen minutes for its performance on the cadaver.

I was fifty minutes completing it on the living subject, as I had to remove a tumor from the point of the temporo-sphenoidal lobe, which delayed me considerably.

---

DR. BRAYTON, editor of the *Indiana Medical Journal*, speaks very highly of calcium sulphydrate as a safe, efficient and economical depilatory. It is best prepared by passing sulphuretted hydrogen gas through thick milk of lime to saturation. The paste is particularly serviceable for operators' use in the preparation of their own hands and the site of operation. The hair grows out again in two or three weeks.

